# Supplementary material for: Transcriptome Analyses of Barley Roots Inoculated with Novel Paenibacillus sp. and Erwinia gerundensis Strains Reveal Beneficial Early-Stage Plant–Bacteria Interactions
Source: Plants (Basel). 2021 Aug 30;10(9):1802. doi: 10.3390/plants10091802 (PMC8467301; doi:10.3390/plants10091802)
Supplement: Supplementary file 1 [file plants-10-01802-s001.zip › Supplementary_tables.pdf]

Supplementary Table S1. Yield (number of clean reads) generated by transcriptome sequencing

	**Sample ID**	**Strain ID**	**Treatment**	**Medium**	**No. of clean reads (Million)**
Bacteria	AR-C-NB-1	AR *Erwinia gerundensis*	control (no barley seedling)	Nutrient Broth	134.4
	AR-C-NB-2				98.7
	AR-C-NB-3				138.6
	AR-B-NB-1		with barley seedlings		121.4
	AR-B-NB-2				145.9
	AR-B-NB-3				106.0
	P25-C-NB-1	S25 *Paenibacillus* sp.	control (no barley seedling)		31.8
	P25-C-NB-2				29.4
	P25-C-NB-3				29.1
	P25-B-NB-1		with barley seedlings		110.3
	P25-B-NB-2				91.6
	P25-B-NB-3				90.5
	P02-C-NB-1	S02 *Paenibacillus* sp.	control (no barley seedling)		26.7
	P02-C-NB-2				30.8
	P02-C-NB-3				41.7
	P02-B-NB-1		with barley seedlings		29.4
	P02-B-NB-2				44.8
	P02-B-NB-3				62.4
	P02-Bu-1		control (no barley seedling)	Burk's N-free	17.7
	P02-Bu-2				26.0
	P02-Bu-3				16.2
	P02-B-Bu-1		with barley seedlings		4.9
	P02-B-Bu-2				22.2
	P02-B-Bu-3				30.1
Plant	B-C-NB-r-1	N/A	control (no bacteria)	Nutrient Broth	155.6
	B-C-NB-r-2				154.5
	B-C-NB-r-3				137.7
	B-C-Bu-r-1			Burk's N-free	122.8
	B-C-Bu-r-2				129.3
	B-C-Bu-r-3				127.9
	B-AR-NB-r-1	AR *Erwinia gerundensis*	with bacteria	Nutrient Broth	146.8
	B-AR-NB-r-2				148.9
	B-AR-NB-r-3				130.4
	B-P25-NB-r-1	S25 *Paenibacillus* sp.			137.9
	B-P25-NB-r-2				93.53
	B-P25-NB-r-3				176.0
	B-P02-NB-r-1	S02 *Paenibacillus* sp.			147.9
	B-P02-NB-r-2				138.7
	B-P02-NB-r-3				165.3
	B-P02-Bu-r-1			Burk's N-free	97.64
	B-P02-Bu-r-2				92.52
	B-P02-Bu-r-3				79.52

Supplementary Table S2. Differentially expressed genes associated with chemotaxis of the three strains when barley seedlings were present

Strain ID	Gene ID	Annotation	Fold change
AR	J9874_00382	methyl-accepting chemotaxis protein	-2.33
	J9874_00704		-2.33
	J9874_01195		-1.93
	J9874_01334		-1.53
	J9874_01896		-1.84
	J9874_03604		-3.00
	J9874_03627		-1.90
	J9874_03746		-1.73
	J9874_01893	chemotaxis protein	-2.17
	J9874_01201	flagellar motor switch protein	1.95
	J9874_01208		5.01
	J9874_01659	sugar ABC transporter permease	1.57
S02	KAI36_01390	methyl-accepting chemotaxis protein	2.18
	KAI36_04010		2.05
	KAI36_03756		1.77
	KAI36_01945		1.73
	KAI36_02050	chemotaxis protein	1.64
	KAI36_04625		2.09
	KAI36_02039		1.60
	KAI36_00103		1.50
	KAI36_02026	flagellar motor switch protein	-2.03
	KAI36_04651	sugar efflux transporter	1.94
S25	KAI37_03945	methyl-accepting chemotaxis protein	3.90
	KAI37_01846		2.96
	KAI37_01348		2.89
	KAI37_02413		2.41
	KAI37_03955		2.25
	KAI37_03695		2.13
	KAI37_03201		1.84
	KAI37_01957	chemotaxis protein	-2.57
	KAI37_04513		-1.77
	KAI37_01947		-1.85
	KAI37_00106		-2.01
	KAI37_01934	flagellar motor switch protein	1.57
	KAI37_04538	sugar efflux transporter	2.17

AR: Novel *E. gerundensis* strain
S02/S25: Novel *Paenibacillus* sp. strains

Supplementary Table S3. Differentially expressed genes associated with biofilm formation of the three strains when barley seedlings were present

**Strain ID**	**Gene ID**	**Annotation**	**Fold change**
AR	J9874_00073	cellulose biosynthesis	1.98
	J9874_03135	glycogen biosynthesis	1.62
	J9874_03136		1.55
	J9874_00235	exopolysaccharide biosynthesis protein	2.77
S02	KAI36_02209	glycogen biosynthesis	-2.62
	KAI36_02210		-2.90
	KAI36_02211		-2.40
	KAI36_01529	exopolysaccharide biosynthesis protein	-1.69
	KAI36_02778		2.70
S25	KAI37_02201	glycogen biosynthesis	17.61
	KAI37_02202		11.66
	KAI37_02709		3.37
	KAI37_01490	exopolysaccharide biosynthesis protein	-2.31

AR: Novel *E. gerundensis* strain
S02/S25: Novel *Paenibacillus* sp. strains

Supplementary Table S4. Differentially expressed genes associated with biofilm formation of *Paenibacillus* sp. S02 compared to *Paenibacillus* sp. S25 when barley seedlings were absent

**Strain ID**	**Gene ID**	**Annotation**	**Fold change**
S02	KAI36_02210	glycogen biosynthesis	39.39
	KAI36_02211		24.74
	KAI36_02209		33.13
	KAI36_02873		5.97
	KAI36_02871		7.05
	KAI36_02778	exopolysaccharide biosynthesis protein	-3.54

S02: Novel *Paenibacillus* sp. strain

Supplementary Table S5. Differentially expressed genes associated with plant growth promotion of strain S02 and S25 when barley seedlings were present

Trait	Gene Name	S02		S25	
**Nitrogen fixation**		**Gene ID**	**Fold change**	**Gene ID**	**Fold change**
	nifB	KAI36_01039	NDE	KAI37_00991	NDE
	nifH	KAI36_01040	-2.84	KAI37_00992	NDE
	nifD	KAI36_01041	-2.48	KAI37_00993	NDE
	nifK	KAI36_01042	-3.37	KAI37_00994	-2.23
	nifE	KAI36_01043	-3.38	KAI37_00995	-1.87
	nifN	KAI36_01044	-4.83	KAI37_00996	-2.18
	nifX	KAI36_01045	-4.79	KAI37_00997	NDE
	hesA/moeB	KAI36_01046	-3.61	KAI37_00998	NDE
	nifV	KAI36_01047	-2.45	KAI37_00999	NDE
**Phosphate solubilization**					
	gcd	KAI36_02793	NDE	KAI37_02644	NDE
**Phosphonate cluster (*phn*)**					
	phnA	KAI36_05309	2.27	KAI37_05177	NDE
	phnB	KAI36_00880	NDE	KAI37_00854	2.75
	phnC	KAI36_04607	NDE	KAI37_04494	-2.78
	phnD	KAI36_04606	-1.54	KAI37_04493	-2.45
	phnE	KAI36_04608	NDE	KAI37_04495	NDE
	phnW	KAI36_05275	-3.31	KAI37_05152	NDE
	phnX	KAI36_00485	NDE	KAI37_00496	NDE
	ppd	KAI36_05276	-2.73	KAI37_05153	NDE
	pepM	KAI36_05277	-2.00	KAI37_05154	NDE
**Phosphate transporter (*pst*)**					
	pstS	KAI36_01689	NDE	KAI37_01595	-5.91
	pstA	KAI36_01691	-1.79	KAI37_01597	-2.30
	pstB	KAI36_01692	-1.52	KAI37_01598	-3.51
	pstC	KAI36_01690	NDE	KAI37_01596	-2.19
	phoP	KAI36_01703	1.73	KAI37_01609	NDE
	phoR	KAI36_01702	1.54	KAI37_01608	2.70
**Indole-3-acetic acid production**					
	ipdC	KAI36_01475	NDE	KAI37_01435	NDE
	auxin efflux carriers	KAI36_02845<br>KAI36_03330<br>KAI36_05253	NDE<br>NDE<br>2.34	KAI37_02683<br>KAI37_03351<br>KAI37_05129	NDE<br>-2.65<br>986.13

S02/S25: Novel *Paenibacillus* sp. strains
NDE: Not differentially expressed

Supplementary Table S6. Differentially expressed genes associated with biological nitrogen fixation of strain S02 when barley seedlings were present (in Burk's N-free medium)

Strain ID	Gene ID	Annotation	Fold change
S02	KAI36_01039	*nifB*	7.34
	KAI36_01040	*nifH*	10.50
	KAI36_01041	*nifD*	11.12
	KAI36_01042	*nifK*	8.54
	KAI36_01043	*nifE*	10.86
	KAI36_01044	*nifN*	6.07
	KAI36_01045	*nifX*	NDE
	KAI36_01046	*hesA/moeB*	NDE
	KAI36_01047	*nifV*	4.13
	KAI36_03691	molybdate-binding protein	4.27
	KAI36_03812		7.22

S02: Novel *Paenibacillus* sp. strain
NDE: Not differentially expressed

Supplementary Table S7. Differentially expressed genes associated with plant growth promotion of strain AR when barley seedlings were present

Strain ID	Gene ID	Annotation	Fold change
AR	J9874_03437	phosphate transporter (*pstS*)	NDE
	J9874_03438	phosphate transporter (*pstC*)	NDE
	J9874_03439	phosphate transporter (*pstA*)	-1.61
	J9874_03440	phosphate transporter (*pstB*)	-1.73
	J9874_03441	phosphate transport system regulator (*phoU*)	NDE
	J9874_02162	auxin efflux carriers	-1.54

AR: Novel *E. gerundensis* strain
NDE: Not differentially expressed

Supplementary Table S8. Differentially expressed core biosynthetic genes of secondary metabolite gene clusters of strain S02 and S25 when barley seedlings were present

**ID**	**Type**	**Most similar known cluster (similarity)**	**S02**		**S25**	
			Gene ID	Fold change	Gene ID	Fold change
C1	Nrps	fusaricidin B (100%)	KAI36_00078	NDE	KAI37_00078	NDE
			KAI36_00083	-2.50	KAI37_00083	-3.44
C2	siderophore	N/A	KAI36_00955	NDE	KAI37_00927	3.71
			KAI36_00956	NDE	KAI37_00928	4.23
			KAI36_00959	1.50	KAI37_00931	NDE
C3	bacteriocin	N/A	KAI36_01103	1.51	KAI37_01049	4.07
C4	Nrps transAT-PKS	N/A	KAI36_01166	-1.75	KAI37_01130	-1.63
			KAI36_01170	-1.82	KAI37_01134	2.35
			KAI36_01172	-2.03	KAI37_01136	NDE
			KAI36_01173	-1.58	KAI37_01137	NDE
			KAI36_01175	NDE	KAI37_01139	NDE
			KAI36_01176	NDE	KAI37_01140	NDE
			KAI36_01178	-2.72	KAI37_01142	NDE
			KAI36_01179	-2.04	KAI37_01143	NDE
			KAI36_01180	-2.52	KAI37_01144	-2.32
			KAI36_01181	-2.17	KAI37_01145	-2.27
C5	lassopeptide	paeninodin (40%)	KAI36_01236	-1.58	KAI37_01200	NDE
			KAI36_01240	-1.69	KAI37_01204	NDE
C6	Nrps	marthiapeptide A (33%)	KAI36_01339	NDE	KAI37_01293	6.70
			KAI36_01340	NDE	KAI37_01294	3.00
			KAI36_01341	-2.23	KAI37_01295	2.61
C7	lanthipeptide	paenilan (100%)	KAI36_01558	NDE	KAI37_01518	NDE
			KAI36_01560	NDE	KAI37_01520	2.73
			KAI36_01562	NDE	KAI37_01522	NDE
C8	lanthipeptide	paenicidin B (71%)			KAI37_01661	-2.25
					KAI37_01663	-1.67
C9	Nrps-like	N/A	KAI36_01944	1.60	KAI37_01854	-1.52
C10	Nrps	tridecaptin (100%)	KAI36_02333	1.55	KAI37_02322	-2.89
			KAI36_02334	NDE	KAI37_02323	NDE
C11	Nrps transAT-PKS	paenilipoheptin (S02, 73%; S25, 76%)	KAI36_02506	2.53	KAI37_02476	12.81
			KAI36_02507	NDE	KAI37_02477	13.60
			KAI36_02508	1.90	KAI37_02478	2.41
			KAI36_02509	2.09	KAI37_02479	1.59
			KAI36_02510	1.74	KAI37_02480	NDE
C12	Nrps	N/A			KAI37_02516	-2.69
C13	Nrps betalactone	N/A			KAI37_02623	NDE
					KAI37_02624	1.87
					KAI37_02633	-1.92
C14	Nrps T3PKS transAT-PKS	aurantinin B/C/D (35%)	KAI36_03362	NDE	KAI37_03372	NDE
			KAI36_03363	NDE	KAI37_03373	NDE
			KAI36_03365	NDE	KAI37_03375	NDE
			KAI36_03366	-2.23	KAI37_03376	NDE
			KAI36_03367	NDE	KAI37_03377	-2.62
			KAI36_03368	NDE	KAI37_03378	-1.90
			KAI36_03371	1.65	KAI37_03381	NDE
			KAI36_03372	NDE	KAI37_03382	-2.77
C15	Nrps	polymyxin (100%)	KAI36_04684	1.57	KAI37_04566	-2.34
					KAI37_04567	-1.63
			KAI36_04687	1.74	KAI37_04570	NDE
			KAI36_04688	1.53	KAI37_04571	NDE
C16	phosphonate	N/A	KAI36_05277	-2.00	KAI37_05154	NDE

S02/S25: Novel *Paenibacillus* sp. strains
NDE: not differentially expressed; Clusters in blue: known antimicrobial compounds

Supplementary Table S9. Differentially expressed core biosynthetic genes of secondary metabolite gene clusters of strain S02 when barley seedlings were present (in Burk's N-free medium)

ID	Type	Most similar known cluster (similarity)	S02	
			**Gene ID**	**Fold change**
C1	Nrps	fusaricidin B (100%)	KAI36_00078	4.94
			KAI36_00083	8.31
C2	siderophore	N/A	KAI36_00955	NDE
			KAI36_00956	NDE
			KAI36_00959	-2.42
C3	bacteriocin	N/A	KAI36_01103	NDE
C4	Nrps<br>transAT-PKS	N/A	KAI36_01166	53.46
			KAI36_01170	236.34
			KAI36_01172	269.16
			KAI36_01173	247.75
			KAI36_01175	244.58
			KAI36_01176	174.31
			KAI36_01178	134.82
			KAI36_01179	79.06
			KAI36_01180	75.55
			KAI36_01181	51.95
C5	lassopeptide	paeninodin (40%)	KAI36_01236	2.96
			KAI36_01240	NDE
C6	Nrps	marthiapeptide A (33%)	KAI36_01339	84.91
			KAI36_01340	60.46
			KAI36_01341	48.96
C7	lanthipeptide	paenilan (100%)	KAI36_01558	7.22
			KAI36_01560	12.29
			KAI36_01562	11.36
C9	Nrps-like	N/A	KAI36_01944	NDE
C10	Nrps	tridecaptin (100%)	KAI36_02333	4.20
			KAI36_02334	5.36
C11	Nrps<br>transAT-PKS	paenilipoheptin<br>(S02, 73%; S25, 76%)	KAI36_02506	NDE
			KAI36_02507	3.21
			KAI36_02508	4.95
			KAI36_02509	7.65
			KAI36_02510	5.29
C14	Nrps<br>T3PKS<br>transAT-PKS	aurantinin B/C/D (35%)	KAI36_03362	4.72
			KAI36_03363	17.63
			KAI36_03365	10.78
			KAI36_03366	8.93
			KAI36_03367	8.27
			KAI36_03368	9.10
			KAI36_03371	4.62
			KAI36_03372	6.49
C15	Nrps	polymyxin (100%)	KAI36_04684	3.80
			KAI36_04687	2.89
			KAI36_04688	2.12
C16	phosphonate	N/A	KAI36_05277	NDE

S02: Novel *Paenibacillus* sp. strain
NDE: Not differentially expressed
Clusters in blue: Known antimicrobial compounds

Supplementary Table S10. Differentially expressed core biosynthetic genes of secondary metabolite gene clusters of strain AR when barley seedlings were present

ID	Type	Most similar known cluster (similarity)	AR	
			Gene ID	Fold change
C1	linear azol(in)e-containing peptides	N/A	J9874_01604<br>J9874_01622	NDE<br>NDE
C2	arylpolyene<br>hserlactone	aryl polyenes (88%)	J9874_01995<br>J9874_01998<br>J9874_02030	NDE<br>NDE<br>NDE
C3	thiopeptide	N/A	J9874_02102<br>J9874_02105	NDE<br>-1.85
C4	Nrps	N/A	J9874_02933<br>J9874_02940	NDE<br>-1.54
C5	terpene	carotenoid (100%)	J9874_03208<br>J9874_03210	1.55<br>2.37
C6	hserlactone	N/A	J9874_03481	NDE
C7	siderophore	N/A	J9874_03599<br>J9874_03601	NDE<br>NDE

AR: Novel *E. gerundensis* strain
NDE: Not differentially expressed

Supplementary Table S11. Differentially expressed transcripts encoding disease resistance proteins and heat shock proteins in barley seedlings when the three bacterial strains were present

Strain ID	Annotation	No. of upregulated transcripts	No. of downregulated transcripts
AR	Disease resistance proteins	84	96
	Heat shock proteins	31	12
S02	Disease resistance proteins	39	41
	Heat shock proteins	12	9
S25	Disease resistance proteins	64	103
	Heat shock proteins	24	17
S02 (in Burk’s N-free medium)	Disease resistance proteins	58	35
	Heat shock proteins	32	6

AR: Novel *E. gerundensis* strain
S02/S25: Novel *Paenibacillus* sp. strains

Supplementary Table S12. Differentially expressed transcripts associated with defence and stress responses in barley seedlings when the three bacterial strains were present

Strain ID	Transcript ID	Annotation	Fold change
AR	BART1_0-p09460.001	leucine-rich receptor kinase	1.75
	BART1_0-p51301.001	polygalacturonase inhibitor	6.36
	BART1_0-p07261.001	xylanase inhibitor	145.89
	BART1_0-p50355.001	caffeoyl CoA *O*-methyltransferase	8.76
	BART1_0-p52030.004		-12.71
	BART1_0-p52031.001		3.49
	BART1_0-p12652.001	glutamate decarboxylase	48.58
	BART1_0-p12655.001		2.80
	BART1_0-p17187.003		2.43
	BART1_0-p17187.004		1.81
	BART1_0-p30229.001		6.77
	BART1_0-p08022.001	ubiquitin-activating enzyme E1	1.89
	BART1_0-p35458.001		1.65
	BART1_0-p35458.005		2.24
	BART1_0-p35458.009		1.61
	BART1_0-p14610.004	ascorbate peroxidase	-40.11
	BART1_0-p14611.002		43.98
	BART1_0-p14611.004		38.10
S02	BART1_0-p59228.001	leucine-rich receptor kinase	-2.87
	BART1_0-p09955.001	polygalacturonase inhibitor	-13.96
	BART1_0-p50356.001	caffeoyl CoA *O*-methyltransferase	-33.51
	BART1_0-p30229.001	glutamate decarboxylase	8.31
	BART1_0-p35458.005	ubiquitin-activating enzyme E1	1.55
	BART1_0-p35458.008		-163.22
	BART1_0-p35458.009		1.61
S25	BART1_0-p09955.001	polygalacturonase inhibitor	-6.43
	BART1_0-p15038.001		-3.57
	BART1_0-p37179.001	caffeoyl CoA *O*-methyltransferase	-2.80
	BART1_0-p52031.001		2.75
	BART1_0-p12655.001	glutamate decarboxylase	-2.31
	BART1_0-p17187.003		2.72
	BART1_0-p17187.005		2.32
	BART1_0-p17187.011		-625.82
	BART1_0-p26684.001		42.12
	BART1_0-p30229.001		-1.85
	BART1_0-p08024.002	ubiquitin-activating enzyme E1	-2.45
	BART1_0-p35458.001		1.75
S02 (in Burk's N-free medium)	BART1_0-p09955.001	polygalacturonase inhibitor	-13.96
	BART1_0-p09460.001	leucine-rich receptor kinase	-1.90
	BART1_0-p59228.001		-6.26
	BART1_0-p37179.001	caffeoyl CoA *O*-methyltransferase	-2.82
	BART1_0-p52030.001		-3.26
	BART1_0-p52030.003		-4.90
	BART1_0-p52031.001		-4.06
	BART1_0-p12654.004	glutamate decarboxylase	37.48
	BART1_0-p12654.006		93.49
	BART1_0-p12655.001		2.93
	BART1_0-p17187.004		-2.14
	BART1_0-p26684.001		-3.17
	BART1_0-p30229.001		4.56
	BART1_0-p08022.001	ubiquitin-activating enzyme E1	-1.74
	BART1_0-p27782.001		-1.62
	BART1_0-p29625.001		-1.70

AR: Novel *E. gerundensis* strain; S02/S25: Novel *Paenibacillus* sp. strains

Supplementary Table S13. Differentially expressed transcripts encoding endoglucanase in barley seedlings when the three bacterial strains were present

Strain ID	Transcript ID	Fold change
AR	BART1_0-p02416.002	2.74
	BART1_0-p13013.001	1.89
	BART1_0-p13221.001	1.56
	BART1_0-p13221.002	7.07
	BART1_0-p13221.003	2.03
	BART1_0-p13224.001	8.98
	BART1_0-p16546.001	-51.59
	BART1_0-p26867.004	-169.13
	BART1_0-p35786.001	-2.70
	BART1_0-p35791.001	138171.33
	BART1_0-p36297.001	134.32
	BART1_0-p36298.001	180.65
	BART1_0-p36300.001	110.53
	BART1_0-p37935.001	2.97
	BART1_0-p37939.001	25.44
	BART1_0-p38044.002	-137.95
	BART1_0-p38044.019	-174.56
	BART1_0-p42923.003	-3.93
	BART1_0-p42924.003	755.12
	BART1_0-p42924.004	1275.61
	BART1_0-p46758.001	1.88
	BART1_0-p50604.001	-198.34
	BART1_0-p50609.001	16.40
	BART1_0-p51464.001	1.50
	BART1_0-p51464.002	2.21
	BART1_0-p59306.001	35.34
S25	BART1_0-p02416.002	2.43
	BART1_0-p13013.001	1.65
	BART1_0-p13059.001	-2.21
	BART1_0-p13221.002	4.85
	BART1_0-p13224.001	15.77
	BART1_0-p29302.001	1.62
	BART1_0-p37930.001	-109.68
	BART1_0-p38044.001	-1.50
	BART1_0-p43284.001	272.20
	BART1_0-p46514.001	-7.18
	BART1_0-p50604.001	-196.79
	BART1_0-p50607.001	-150.94
	BART1_0-p51464.002	3.10
	BART1_0-p53733.001	-6.40
	BART1_0-p55128.003	-1.60
	BART1_0-p59304.001	-62.31
S02	BART1_0-p05997.001	-3.67
	BART1_0-p19258.001	-2.11
	BART1_0-p38044.001	-1.55
	BART1_0-p50607.001	-12.19
	BART1_0-p53733.001	-2.32
	BART1_0-p55128.003	-1.55
S02 (in Burk's N-free medium)	BART1_0-p02416.002	2.02
	BART1_0-p05997.001	-13.56
	BART1_0-p13059.001	-5.57
	BART1_0-p13221.001	-2.83
	BART1_0-p13221.003	-1.89
	BART1_0-p16188.001	-13.24
	BART1_0-p19258.001	-8.17
	BART1_0-p35786.001	-15.32
	BART1_0-p37930.001	-30.63
	BART1_0-p38044.001	-1.70
	BART1_0-p43284.001	260.24
	BART1_0-p46514.001	-3.19
	BART1_0-p46758.001	-1.60
	BART1_0-p51464.001	-2.16
	BART1_0-p51466.001	-2.49
	BART1_0-p53733.001	-6.94
	BART1_0-p55128.001	2.45

AR: Novel *E. gerundensis* strain
S02/S25: Novel *Paenibacillus* sp. strains

Supplementary Table S14. Differentially expressed transcripts associated with signal transduction and ethylene biosynthesis in barley seedlings when the three bacterial strains were present

Strain ID	Transcript ID	Annotation	Fold change
AR	BART1_0-p22808.001	ADP-ribosylation factor	2.03
	BART1_0-p32313.002		10670.18
	BART1_0-p32313.003		4579.19
	BART1_0-p32313.004		244.17
	BART1_0-p40410.001		1.58
	BART1_0-p59482.001		1.98
	BART1_0-p59482.003		1.89
	BART1_0-p27744.001	GTP-binding proteins	-57.23
	BART1_0-p35339.001		310.46
	BART1_0-p36228.006		-2.52
	BART1_0-p38804.001		1.60
	BART1_0-p38805.001		78.48
	BART1_0-p38805.002		138.92
	BART1_0-p27326.001	aminocyclopropanecarboxylate oxidase	-72.03
	BART1_0-p27327.002		-2.83
S02	BART1_0-p22808.001	ADP-ribosylation factor	1.56
	BART1_0-p22808.009		1.72
	BART1_0-p27743.001	GTP-binding proteins	3.88
	BART1_0-p36228.006		-1.84
	BART1_0-p27326.001	aminocyclopropanecarboxylate oxidase	-3.70
	BART1_0-p27327.001		2.64
S25	BART1_0-p22808.001	ADP-ribosylation factor	1.75
	BART1_0-p42864.001		1.61
	BART1_0-p59482.001		1.63
	BART1_0-p59482.003		1.66
	BART1_0-p59482.006		2.86
	BART1_0-p27743.001	GTP-binding proteins	2.53
	BART1_0-p38805.001		19.51
	BART1_0-p27326.001	aminocyclopropanecarboxylate oxidase	-314.69
	BART1_0-p27327.002		-15.25
S02 (in Burk's N-free medium)	BART1_0-p40410.004	ADP-ribosylation factor	-30.77
	BART1_0-p59482.002		26.67
	BART1_0-p59482.003		1.61
	BART1_0-p36228.001	GTP-binding proteins	-1.79
	BART1_0-p27326.001	aminocyclopropanecarboxylate oxidase	-9.00

AR: Novel *E. gerundensis* strain
S02/S25: Novel *Paenibacillus* sp. strains

Supplementary Table S15. Differentially expressed transcripts encoding high affinity transporters in barley seedlings when the three bacterial strains were present

**Strain ID**		**Transcript ID**	**Fold change**
AR	nitrate	BART1_0-p42069.001	-92.78
		BART1_0-p42070.001	-324.24
		BART1_0-p42072.001	9.36
		BART1_0-p42073.001	-41.16
		BART1_0-p42077.001	128.40
		BART1_0-p42082.001	41.67
		BART1_0-p55377.001	-80.43
		BART1_0-p40717.001	-10.69
		BART1_0-p45131.001	-5.72
	iron	BART1_0-p53866.001	1.83
	potassium	BART1_0-p14481.001	8.05
		BART1_0-p14481.002	8.40
		BART1_0-p14476.001	2.46
		BART1_0-p59178.002	37.21
	sulphate	BART1_0-p30667.002	-93.09
		BART1_0-p30667.004	-12.07
		BART1_0-p30667.005	-18.58
		BART1_0-p30667.006	-493.77
		BART1_0-p30667.007	-37.07
S25	nitrate	BART1_0-p42069.001	-126.57
		BART1_0-p42070.001	-478.53
		BART1_0-p42072.001	-17.42
		BART1_0-p42073.001	-123.42
		BART1_0-p42079.001	-321.70
		BART1_0-p42084.001	-2.22
		BART1_0-p42092.001	-5.27
		BART1_0-p42093.001	-6.37
		BART1_0-p55377.001	-201.97
		BART1_0-p40717.001	-11.20
		BART1_0-p45131.001	-4.93
	potassium	BART1_0-p14481.001	19.52
		BART1_0-p14481.002	19.05
		BART1_0-p14476.001	4.60
		BART1_0-p59179.001	4.76
	sulphate	BART1_0-p30667.002	-9.51
		BART1_0-p30667.005	-5.13
	inorganic phosphate	BART1_0-p31346.001	-514.99
		BART1_0-p31572.001	-33.95

**Strain ID**		**Transcript ID**	**Fold change**
S02	nitrate	BART1_0-p40717.001	-1.69
		BART1_0-p42069.001	-150.75
		BART1_0-p42070.001	-71.16
		BART1_0-p42073.001	-9.55
		BART1_0-p42079.001	2.65
		BART1_0-p42084.001	-7.89
		BART1_0-p42092.001	-31.39
		BART1_0-p42093.001	-11.37
		BART1_0-p45131.001	-2.35
		BART1_0-p40717.001	-1.69
	iron	BART1_0-p53866.001	1.80
	potassium	BART1_0-p59179.001	-1.72
	sulphate	BART1_0-p30667.002	-8.88
		BART1_0-p30667.004	-3.70
		BART1_0-p30667.005	-4.61
		BART1_0-p30670.001	3.29
	inorganic phosphate	BART1_0-p31346.001	-15.73
		BART1_0-p31572.001	-18.34
S02 (in Burk' s N-free medium)	nitrate	BART1_0-p42069.001	-193.11
		BART1_0-p42070.001	-97.43
		BART1_0-p42078.001	-2.13
		BART1_0-p42079.001	-909.93
		BART1_0-p42084.001	-650.54
		BART1_0-p42092.001	-983.39
		BART1_0-p42093.001	-143.76
		BART1_0-p45131.001	-2.53
		BART1_0-p45134.001	-3.99
	potassium	BART1_0-p14476.001	-2.48
		BART1_0-p59179.001	4.17
	sulphate	BART1_0-p30667.002	-30.78
		BART1_0-p30667.004	-3.03
		BART1_0-p30668.001	3.26
		BART1_0-p30670.001	-12.50

AR: Novel *E. gerundensis* strain
S02/S25: Novel *Paenibacillus* sp. strains

Supplementary Table S16. Differentially expressed transcripts associated with nutrient uptake and metabolism in barley seedlings when the three bacterial strains were present

Strain ID	Transcript ID	Annotation	Fold change
AR	BART1_0-p04828.001	ammonium transporter	-457.99
	BART1_0-p04829.001		160.67
	BART1_0-p40957.001		-7.40
	BART1_0-p45407.001		-3.08
	BART1_0-p58582.001		-4.47
	BART1_0-p01562.001	anthocyanidin-*O*-glucosyltransferase	91.46
	BART1_0-p01563.001		2.11
	BART1_0-p40352.001		-2.30
	BART1_0-p50969.002		-6.28
	BART1_0-p38221.001		1.82
	BART1_0-p38224.001		174.97
	BART1_0-p22047.004		1063.42
	BART1_0-p26633.001	glutamine synthetase	4.49
	BART1_0-p26633.002		2.51
	BART1_0-p26633.003		6.65
	BART1_0-p26633.004		3.03
	BART1_0-p46549.002		1.89
	BART1_0-p46549.007		1.65
	BART1_0-p22331.001	aspartate aminotransferase	4.74
	BART1_0-p22331.002		2.16
	BART1_0-p22331.008		5.06
	BART1_0-p22331.011		23.73
	BART1_0-p22331.013		67.08
	BART1_0-p48209.001	sucrose synthase	2.80
	BART1_0-p48951.001		4.61
	BART1_0-p50918.003		2.69
	BART1_0-p50918.004		2.33
	BART1_0-p50919.002		2.20
	BART1_0-p04906.023	UDP-glucose pyrophosphorylase	753.55
	BART1_0-p04906.031		2.71
S02	BART1_0-p04827.001	ammonium transporter	2.30
	BART1_0-p40957.001		-3.21
	BART1_0-p58582.001		-2.59
	BART1_0-p22331.001	aspartate aminotransferase	2.69
	BART1_0-p22331.002		1.73
	BART1_0-p54667.001		1.92
	BART1_0-p58839.006		3.27
	BART1_0-p58839.015		43.44
	BART1_0-p09325.001	sucrose synthase	-3.05
	BART1_0-p50918.003		1.77
	BART1_0-p50918.013		2.35
	BART1_0-p50919.003		-2.15
	BART1_0-p50919.018		1.72

S25	BART1_0-p04828.001	ammonium transporter	-5.66
	BART1_0-p40957.001		-8.34
	BART1_0-p45407.001		-4.09
	BART1_0-p58582.001		-3.34
	BART1_0-p26633.001	glutamine synthetase	5.72
	BART1_0-p26633.002		6.38
	BART1_0-p26633.003		15.22
	BART1_0-p26633.004		6.16
	BART1_0-p46549.001		1.92
	BART1_0-p46549.002		3.87
	BART1_0-p46549.007		2.84
	BART1_0-p46549.008		2.77
	BART1_0-p46549.010		3.18
	BART1_0-p22331.001	aspartate aminotransferase	3.56
	BART1_0-p22331.002		1.57
	BART1_0-p22331.007		4.84
	BART1_0-p22331.008		6.08
	BART1_0-p22331.011		12.17
	BART1_0-p58839.001		-2.42
	BART1_0-p58839.015		57.59
	BART1_0-p48209.001	sucrose synthase	1.88
	BART1_0-p48951.001		4.45
	BART1_0-p48951.009		4.64
	BART1_0-p50918.013		3.53
	BART1_0-p50918.016		30.21
	BART1_0-p50919.002		2.70
S02 (in Burk's N-free medium)	BART1_0-p23459.001	ammonium transporter	-3.47
	BART1_0-p40957.001		-9.80
	BART1_0-p45407.001		-16.10
	BART1_0-p46549.007	glutamine synthetase	2.37
	BART1_0-p22331.001	aspartate aminotransferase	3.14
	BART1_0-p22331.002		1.91
	BART1_0-p22331.011		3.57
	BART1_0-p54667.001		-2.16
	BART1_0-p55159.001		7.04
	BART1_0-p50918.004	sucrose synthase	-2.12
	BART1_0-p50918.013		1.90
	BART1_0-p50919.017		30.36

AR: Novel *E. gerundensis* strain
S02/S25: Novel *Paenibacillus* sp. strains
